# Dental Caries and Oral Mucositis Among Children Undergoing Cancer Therapy at Kenyatta National Hospital, Kenya: A Cross-Sectional Study

**DOI:** 10.7759/cureus.94417

**Published:** 2025-10-12

**Authors:** Diana A Okello, Mary Masiga, Marjorie Muasya

**Affiliations:** 1 Paediatric Dentistry, Department of Dental Sciences, University of Nairobi, Nairobi, KEN

**Keywords:** cancer therapy, dental caries, methotrexate, oncology, oral mucositis

## Abstract

Background

Dental caries and oral mucositis are often experienced by children undergoing antineoplastic therapy. Understanding the prevalence and characteristics of these complications is essential for developing targeted supportive oral care strategies to improve overall treatment outcomes and the quality of life of the children.

Methods

This study employed a cross-sectional design with 102 children aged three to 12 years undergoing cancer therapy at Kenyatta National Hospital, Kenya. Participants were selected through purposive sampling. Data was collected via a structured questionnaire and clinical oral examination, with dental caries assessed using the decayed, missing, and filled teeth (dmft, primary teeth)/Decayed, Missing, and Filled Teeth (DMFT, permanent teeth) index, and oral mucositis assessed using the WHO Oral Mucositis Scale (1979).

Results

Acute lymphoblastic leukaemia was the most prevalent cancer subtype and chemotherapy the most common antineoplastic treatment. The prevalence of dental caries was 58.8%, with higher occurrence in children aged six to 12 years (p = 0.025). Oral mucositis was observed in 28.4% of children, mainly Grades I and II, while only 2.9% had undergone dental assessment before cancer therapy.

Discussion

Children undergoing cancer therapy had a higher prevalence of dental caries compared to the 2015 Kenya National Oral Health Survey. Oral mucositis prevalence was lower than many reports, likely reflecting study design and patient heterogeneity.

Conclusion

Including a dentist in the multidisciplinary cancer care team may improve outcomes through pre-treatment assessments, ongoing oral care and active surveillance of oral complications due to antineoplastic treatment.

## Introduction

Approximately 80% of global childhood cancer cases occur in low- and middle-income countries (LMICs), a disparity primarily attributed to the predominance of younger populations within these regions [1]. The management of childhood cancers involves multiple therapeutic modalities, including chemotherapy, radiotherapy, surgery, and, more recently, haematopoietic stem cell transplantation (HSCT) and immunotherapy [2-4]. These treatment options are frequently associated with oral complications, such as dental caries, oral infections, oral mucositis, and xerostomia [5]. These oral complications can significantly affect the nutritional status and overall health, including the quality of life of paediatric oncology patients [6,7]. Consequently, a thorough understanding of the regional paediatric cancer burden and the prevalence of therapy-related oral morbidities is essential for informing effective, context-specific supportive care strategies that enhance treatment outcomes and patient well-being.

Chemotherapy is associated with oral complications in approximately 40% of patients, with the prevalence rising to over 90% in children under the age of 12 years [7-9]. Eighty to 100% of patients receiving radiotherapy directed to the head and neck region inevitably develop radiation-induced mucositis, and the addition of chemotherapy significantly increases this risk [10-12]. Approximately 80% of children undergoing HSCT experience oral mucositis, xerostomia, dysgeusia, as well as both local and systemic infections [13,14]. The most frequently reported oral toxicities linked with immunotherapeutic agents include low-grade mucositis, lichenoid mucosal reactions, dysgeusia, and xerostomia [15,16]. Despite these known complications, data on the immunotherapy-induced oral adverse events in paediatric patients remains scarce.

Several studies examining the oral complications of antineoplastic therapy have identified dental caries and oral mucositis as the most prevalent conditions. Dental caries is a non-communicable biofilm-driven disease, influenced by prolonged exposure to cariogenic dietary habits, resulting in the progressive demineralization of tooth structure [17]. This condition is largely preventable, and its prevention plays a vital role in the oral healthcare of paediatric patients undergoing cancer treatment [18-20]. Wang et al. investigated 39 children diagnosed with acute lymphoblastic leukemia (ALL) undergoing chemotherapy and compared them with age-matched counterparts who were not diagnosed with ALL. This Chinese study reported that 69.2% of all documented oral conditions were dental caries. This high prevalence was linked to the methotrexate’s mucosal cytotoxic effects, which compromised oral hygiene. Moreover, chemotherapy-induced nausea and vomiting often result in children consuming smaller, more frequent meals, which increases the risk of dental caries due to extended exposure of the teeth to dietary sugars. Microbiological analysis of supragingival plaque revealed a higher abundance of Lactobacillus species in the chemotherapy group, further predisposing these children to caries progression. Key contributing factors therefore included frequent eating, poor oral hygiene, shifts in the oral microbiome, and high sugar consumption [21].

Oral mucositis (OM) is characterized by inflammation of the oral mucosa, which is a widespread and often debilitating complication of antineoplastic therapy [7]. Younger age, neutropenia, poor oral hygiene, compromised nutritional status, and the type of malignancy, often influence the development of OM [8]. OM usually develops within five to seven days after starting chemotherapy and can continue throughout the treatment duration, a pattern observed by Cheng et al. in China [22]. Ulcerations generally emerge within seven to 10 days, often presenting with minimal inflammatory infiltration at the margins and requiring up to 14 days to heal. In severe cases, individual lesions can coalesce into large, shallow ulcers with necrotic bases [23-25]. Radiation-induced mucositis (RIM), on the other hand, usually develops around 14 days after the start of radiotherapy and resolves within three to four weeks. Clinically, RIM is marked by ulcers with poorly defined borders and lacks the erythematous halo commonly seen in chemotherapy-induced mucositis. RIM predominantly affects the floor of the mouth, buccal mucosa, and soft palate, while sparing the gingiva and tongue [26]. These painful lesions can severely impair nutrition, oral hygiene practices, and commonly increase susceptibility to secondary infections.

These oral complications significantly contribute to the overall disease burden among paediatric cancer patients. Addressing the current knowledge gap on the prevalence of dental caries and oral mucositis in Kenyan children undergoing cancer therapy, the findings of this study aim to enrich the existing body of literature and provide foundational data to guide future research and inform context-specific clinical management strategies.

## Materials and methods

Study design

Ethical approval to conduct the study was granted by the Kenyatta National Hospital (KNH) Ethics and Research Committee at University of Nairobi (Ref. No. P796/10/2021). This study employed descriptive cross-sectional design at KNH. KNH provides chemotherapy, surgery, and radiotherapy as its main cancer treatment modalities; however, HSCT and immunotherapy were not available during data collection. The hospital has an official bed capacity of approximately 1,800, including 400 pediatric beds, although actual occupancy often exceeds 3,000 due to high patient demand. Paediatric oncology patients are admitted across multiple inpatient wards, with an average of 140 children receiving cancer treatment.

Patient recruitment

Considering the study design, the sample size was determined using Cochran’s formula (Z test) and computed as follows [27]: 

Where n = sample size; Z = value from the standard normal distribution for 95% confidence level = 1.96; p = prevalence of dental caries and oral mucositis among paediatric oncology patients = 0.5; and d = allowable error (absolute) = 0.05.

Therefore, nonetheless, the sample size calculated is for a study population >10,000 and the desired sample size is for a study population <10,000, the sample size was corrected for a study population <10000:

Where n = desired sample size for a study population <10000; n_0_ = sample size derived for a study population >10000; and N = estimated size of the study population (patients) = 140.

$n= n_0/(1+((n_0- 1))/N)$

$n=384/(1+((384-1))/140)$

n=102.

Sample size calculation and finite population correction (FPC) for a paediatric oncology cohort (N=140). Applying the FPC reduced the large-population estimate to achieve the sample size of 102, with a negligible effect on precision.

Therefore, a sample size of 102 paediatric oncology children were enrolled into the study using purposive sampling, based on their eligibility and availability during the study period. Children aged three to 12 years undergoing cancer treatment at the study site were eligible if parental or guardian consent was obtained, with assent provided by the child when appropriate. Children who were critically ill or in medical isolation at the time of recruitment were excluded.

Data collection

Data was collected using two primary methods: a structured questionnaire (Appendix 1) developed by the principal investigator (PI) and a clinical examination (Appendix 2). The questionnaire was adapted and modified from the World Health Organization (WHO) Simplified Oral Health Questionnaire for Children to include a section on medical history, and was pre-tested on 10% of the target sample prior to the main study [28]. Parents or guardians served as the informants for the sections on dietary habits and oral hygiene practices. The ward nurse thereafter prepared the list of all eligible children, and intraoral examinations were subsequently conducted by the PI without prior access to individual medical histories or treatment regimens.

The clinical examinations were conducted by the PI under field conditions using natural light. Each child underwent an intraoral assessment to evaluate the overall oral hygiene status and the presence of oral mucositis and dental caries. Dental caries was assessed by visual-tactile examination using the WHO Oral Health Assessment Form for Children (2013) [28]. Caries experience was recorded with the decayed, missing, and filled teeth (dmft) index for primary teeth and the Decayed, Missing, and Filled Teeth (DMFT) index for permanent teeth, applied as appropriate in mixed dentition, in accordance with WHO Oral Health Survey criteria [28]. Teeth were dried with gauze before inspection. Caries was identified by the presence of white chalky lesions in the cervical region, visible loss of tooth structure, or evidence of previous treatment, such as restorations or extractions. Oral mucositis was evaluated by gently retracting the lips and buccal tissues, with its presence and severity assessed according to the WHO Oral Mucositis Scale (1979) [29]. Oral hygiene was assessed based on dental plaque accumulation. Each child chewed a disclosing tablet for one minute and spread the dye using the tongue. After rinsing, plaque was assessed on six index teeth following the Sillness and Löe criteria [30]. The extent of plaque on buccal and lingual surfaces was quantified using the modified Quigley-Hein Plaque Index by Turesky et al. [31].

The medical history was subsequently obtained by the PI from hospital records and included details such as the child’s age, sex, type of cancer, diagnostic approach, cancer therapy regimen, and any preceding dental care received. This ensured that the PI was blinded during the clinical assessments, thereby reducing the risk of diagnostic bias.

To ensure diagnostic consistency, one of the study supervisors (a senior paediatric dentist) conducted a calibration exercise with the PI on 10% of the sample, standardizing the diagnostic assessment of dental caries, oral mucositis, and dental plaque in one of the paediatric oncology wards. To evaluate both inter-examiner and intra-examiner reliability, Cohen’s Kappa (κ) coefficient was employed. Kappa values for dental caries, oral mucositis, and plaque were 0.82, 0.90, and 0.86, indicating substantial inter-examiner agreement. Intra-examiner reliability was also high with Kappa values of 0.90, 0.96, and 0.85 for dental caries, oral mucositis, plaque scores, demonstrating strong consistency and reproducibility of the PI’s assessments.

Statistical analysis

Data analysis was performed with IBM SPSS version 25 (IBM Corp., Armonk, NY, USA). Continuous variables were summarized using descriptive statistics such as means, standard deviations, and medians. Relationships between variables were examined through Analysis of Variance (ANOVA), Pearson’s Chi-square test, and Spearman’s rank-order correlation. A 95% confidence interval (CI) was applied to determine the reliability and accuracy of the associations. Findings were displayed in tables to enhance and support the narrative.

## Results

Study participant characteristics

One hundred and two participants completed the study, comprising 55 males (53.9%) and 47 females (46.1%). The mean age was 6.08 ± 3.1 years, ranging from three to 12 years. Leukaemia was the most frequently diagnosed malignancy, accounting for 35 cases (34.3%). The majority (59.8%, N=61) received chemotherapy alone. ALL was the most prevalent cancer subtype. Chemotherapy agents, including actinomycin-D, carboplatin, cytarabine, daunorubicin, doxorubicin, etoposide, L-asparaginase, methotrexate, and vincristine, were administered in different combinations and regimens tailored to their specific diagnoses.

Dental caries

For clinical evaluation, participants were grouped by dentition development into three to five years (primary dentition) and six to 12 years (mixed dentition or permanent dentition). Notably, only 2.9% of the participants had received a dental assessment prior to the cancer treatment. A total of 57 children (55.9%) were in the primary dentition phase (ages three to five), while 45 children (44.1%) were in the mixed or permanent dentition phase (ages six to 12).

Dental caries experience was evaluated using the dmft and DMFT indices, categorized according to the stage of dentition. Dental caries prevalence was 58.8%. Among children aged three to five years (primary dentition), the prevalence was 49.1%, whereas a notably higher rate of 71.1% was observed in those aged six to 12 years (mixed or permanent dentition). There was a statistically significant relationship between age and the occurrence of dental caries, with children aged between six and 12 years having a higher odds of dental caries compared to those the younger group (χ² = 5.020, p = 0.025). In the primary dentition group, the average dmft score was 2.33, whereas the mixed/permanent dentition group recorded a 2.78 mean DMFT score. Specifically, permanent teeth alone had a 0.33 mean DMFT score. In both dentition groups, the decayed component constituted the largest proportion of the dmft and DMFT indices, suggesting that most of the carious lesions remained untreated, particularly within the primary dentition, as shown in Table 1.

**Table 1:** Dental caries experience M+SD represents Mean + Standard Deviation; dmft (decayed, missing, filling, teeth [primary]); DMFT, (Decayed, Missing, Filling, Teeth [permanent]). Statistically significant results with p value ≤0.05. The decayed component constituted the largest proportion of the dmft and DMFT indices, suggesting that most of the carious lesions remained untreated, particularly within the primary dentition.

Characteristic	Category	d	m	f	dmft	ANOVA
		M +SD	M +SD	M+SD	M+SD	
Gender	Male	3.02 +3.76	0.0	0.0	3.02+3.76	F(1,100) = 2.38, p=0.126
	Female	1.94 +3.07	0.0	0.02+0.15	1.96+3.08	
Characteristic	Category	D	M	F	DMFT	ANOVA
		M+SD	M+SD	M+SD	M +SD	
Gender	Male	0.13 +0.61	0.04+0.27	0.00	0.16+0.66	F(1,100) = 0.67, p=0.796
	Female	0.06+0.32	0.0	0.06+0.44	0.13+0.74	

Dental caries prevalence was further assessed by cancer therapy type. Children receiving chemotherapy demonstrated a higher dental caries prevalence; however, this observation may be influenced by the fact that chemotherapy was the predominant treatment modality among study participants (Table 2).

**Table 2:** Dental caries prevalence by cancer treatment modalities Statistical significant results with p value ≤0.05. CT – Chemotherapy , RT – Radiotherapy. This table presents the distribution of dental caries among the children receiving cancer treatment.

Characteristic	Category	Caries prevalence				Pearson’s Chi-Square		
		Present		Absent		χ^2^	df	p-value
		n	%	n	%			
Treatment modalities	CT alone	35	57.4	26	42.6	0.992	5	0.963
	CT and RT	4	57.1	3	42.9			
	RT alone	1	100	0	0.0			
	CT and Surgery	14	58.3	10	41.7			
	Surgery alone	2	66.7	1	33.3			
	CT, RT, & Surgery	4	66.7	2	33.3			
	Overall	60	58.8	42	41.2			

Oral mucositis

Oral mucositis overall prevalence was 28.4%. Regarding gender, male children exhibited a slightly higher prevalence than female children. In terms of age, the younger age group (three to five years) demonstrated a higher occurrence of oral mucositis, although the difference was not statistically significant as presented in Table 3.

**Table 3:** Oral mucositis relationship with and age and gender Statistical significant results with p-value ≤0.05

Variable Category		Oral mucositis				Test		
		Present		Absent				
		n	%	n	%	χ^2^	df	p-value
Gender	Male	16	29.1	39	70.9	0.026	1	0.873
	Female	13	27.7	34	72.3			
Age categories (years)	3 – 5	15	26.3	42	73.7	0.284	1	0.594
	6 – 12	14	31.1	31	68.9			

As detailed in Table 4, the majority of children diagnosed with OM were classified as having Grade I or Grade II severity. Specifically, 14 children (13.7%) exhibited Grade II mucositis, 13 children (12.7%) presented with Grade I, and only two children (2%) were identified with Grade III OM.

**Table 4:** The distribution of oral mucositis severity Statistical significant results with p-value ≤0.05

Oral Mucositis Grades	Age groups years				Test	
	3 - 5 (N=57)		6 - 12 (N=45)		χ^2 ^(df)	p-value
	N	%	N	%		
Grade 0 Grade I Grade II Grade III Grade IV	42	57.5%	31	42.5%	3.514 (3)	0.319
	6	46.2%	7	53.8%		
	9	64.3%	5	35.7%		
	0	0.0%	2	100.0%		
	0	0.0%	0	0.0%		

Oral hygiene status

Oral hygiene was evaluated by examining dental plaque presence and severity. All 102 participants (100%) exhibited varying levels of plaque. To measure this, the modified version of the Quigley-Hein Plaque Index by Turesky et al. was applied to assess plaque accumulation on both the buccal and lingual tooth surfaces. Based on the proportion of tooth surfaces covered by plaque, oral hygiene was classified as mild in 12 children (11.8%), moderate in 57 children (55.9%), and severe in 33 children (32.2%). Children receiving a combined a treatment of chemotherapy and surgery exhibited poorer oral hygiene.

## Discussion

Children undergoing cancer treatment frequently experience adverse oral effects, with dental caries and oral mucositis among the most commonly reported conditions [32]. Although ALL comprised the largest subgroup in our cohort, other malignancies were represented; accordingly, interpretations are framed for the overall paediatric oncology population rather than a single diagnosis. At KNH, the standard ALL protocol spans two to three years across five phases (Induction, Consolidation, Interim Maintenance, Delayed Intensification, Maintenance), and most participants were assessed during induction when hospitalization for diagnostic work-up and stabilization is common [33]. Chemotherapeutic agents administered, such as actinomycin-D, carboplatin, cytarabine, daunorubicin, doxorubicin, etoposide, L-asparaginase, methotrexate, and vincristine, were consistent with risk-adapted regimens used globally [34].

Dental caries prevalence in the study was 58.8%, considerably higher than 23.9% reported in the 2015 Kenya National Oral Health Survey [35]. Similar trends were observed in Sudan, where children receiving cancer treatment exhibited a 37.9% prevalence, compared to 24% in the general paediatric population [36]. This reinforces existing evidence that children with systemic illnesses face a disproportionate burden of oral diseases, with oral healthcare being among their most frequently unmet needs [37]. Several chemotherapeutic agents are associated with reduced salivary flow and qualitative salivary alterations that may increase dental caries risk; however, sialometry was not performed in this study, references to hyposalivation are provided as literature context rather than causal inferences from our data [38]. In this cohort, limited access to paediatric dental care evidenced by only 2.9% receiving a dental evaluation during therapy was identified as a system-level risk factor for unmet preventive and therapeutic oral care.

Oral mucositis was present in 28.4% of participants, predominantly Grades I - II, lower than rates reported in prospective cohorts, such as 40% in a Brazilian paediatric cohort and 69% in Swedish children with ALL [9,39]. Differences likely reflect study design and timing in regards clinical assessment. Our cross-sectional assessment provides a point estimate at a single time point and cannot capture onset, peak severity, or fluctuations across treatment cycles. Heterogeneity in diagnoses and treatment phases, including participants assessed during rest periods, may also have allowed for mucosal healing, contributing to lower prevalence. In addition, KNH protocols which provide for prophylactic povidone-iodine mouth rinses and “magic mouthwash” containing lidocaine, chlorpheniramine, dexamethasone, and relcer gel, may mitigate mucosal inflammation, although we cannot ascribe causality [40]. Under detection of oral mucositis may be because daily monitoring was not conducted.

Oral hygiene was generally poor, with plaque observed in all children; among those with oral mucositis, 89.7% had moderate-to-severe plaque. Poor oral hygiene may be attributable to pain or interruptions in routine oral care (e.g. tooth brushing) during hospitalization both frequently reported in children with chronic illnesses [21]. These are descriptive findings from a cross-sectional snapshot and were not intended to test a plaque-oral mucositis association but they should be viewed as hypothesis-generating for future longitudinal studies.

## Conclusions

In this single-centre cross-sectional study of 102 children undergoing cancer therapy at KNH, the prevalence of dental caries was 58.8% and oral mucositis 28.4% (predominantly Grades I - II), with majority having poor oral hygiene and minimal access to dental care (2.9% received a dental evaluation). The latter represents a system-level risk factor and underscores the need to integrate paediatric dental services into oncology pathways e.g., standardized pre-treatment oral screening, routine dental and oral mucositis surveillance, and timely referral, particularly in LMICs.

These findings should be interpreted in light of key limitations such as the cross-sectional design, single-centre, and clinical examinations under field conditions without adjunctive imaging (possible underestimation of early lesions). Future multi-centre, prospective studies with serial oral mucositis grading, objective salivary measures (e.g., sialometry), are recommended. Health ministries and oncology programs should mandate standardized pre-treatment oral screening and risk stratification for all paediatric cancer patients, with embedded referral pathways to paediatric dentistry and routine oral mucositis surveillance documented in the oncology record.

## Figures and Tables

**Figure 1:**
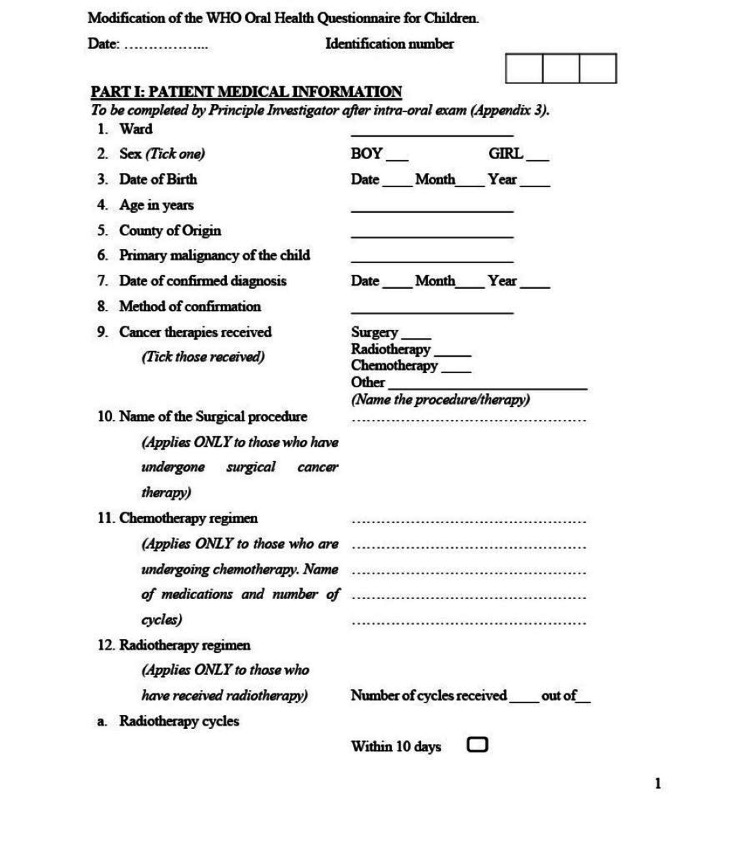
WHO Oral Health Questionnaire page 1 Modified from WHO Oral Health Assessment form for Children, 2013 [28]

**Figure 2:**
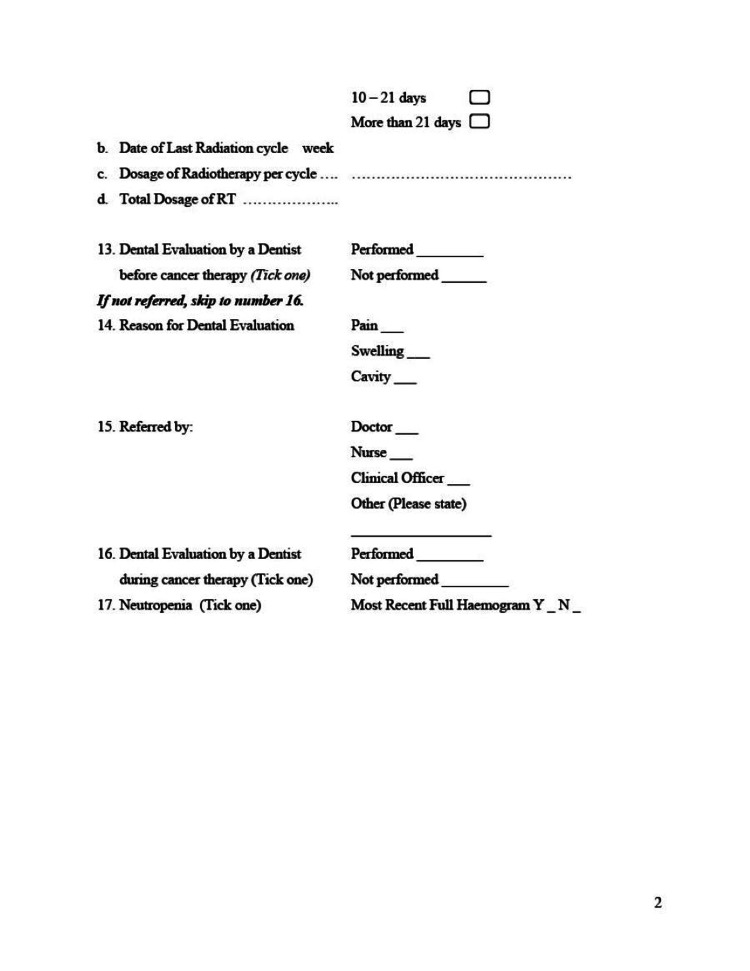
WHO Oral Health Questionnaire page 2 Modified from WHO Oral Health Assessment form for Children, 2013 [28]

**Figure 3:**
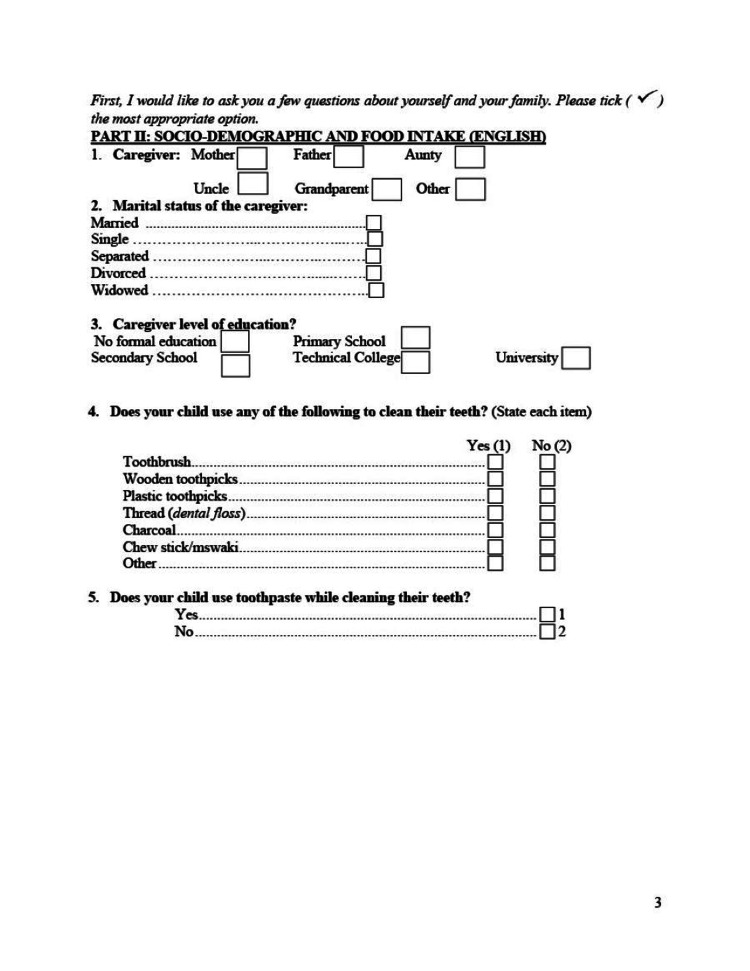
WHO Oral Health Questionnaire page 3 Modified from WHO Oral Health Assessment form for Children, 2013 [28]

**Figure 4:**
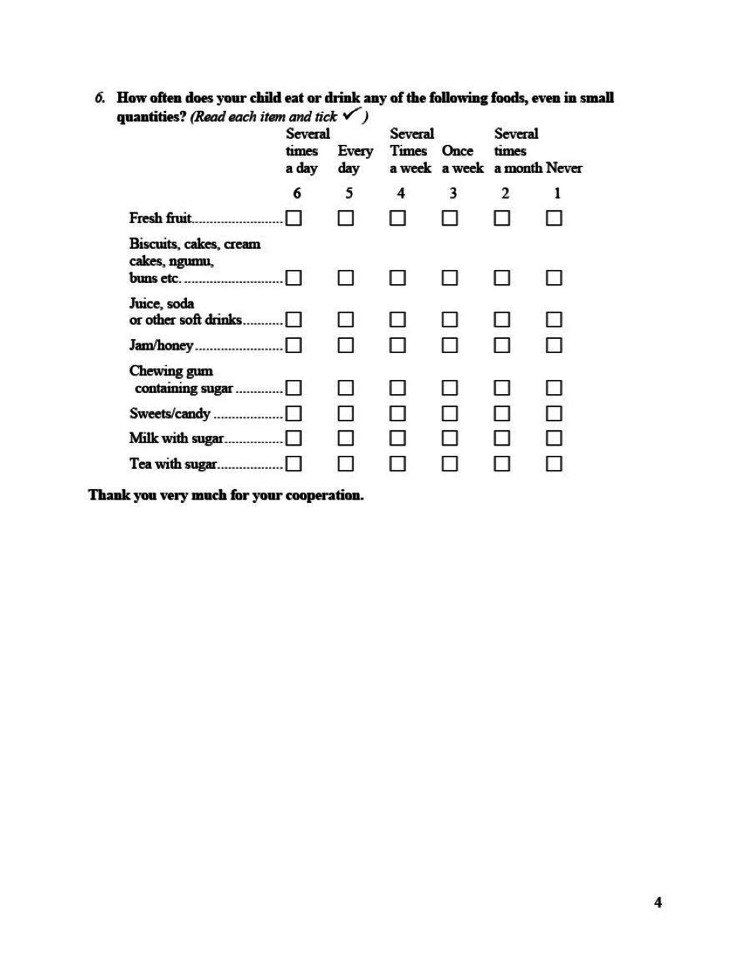
WHO Oral Health Questionnaire page 4 Modified from WHO Oral Health Assessment form for Children, 2013 [28]

**Figure 5:**
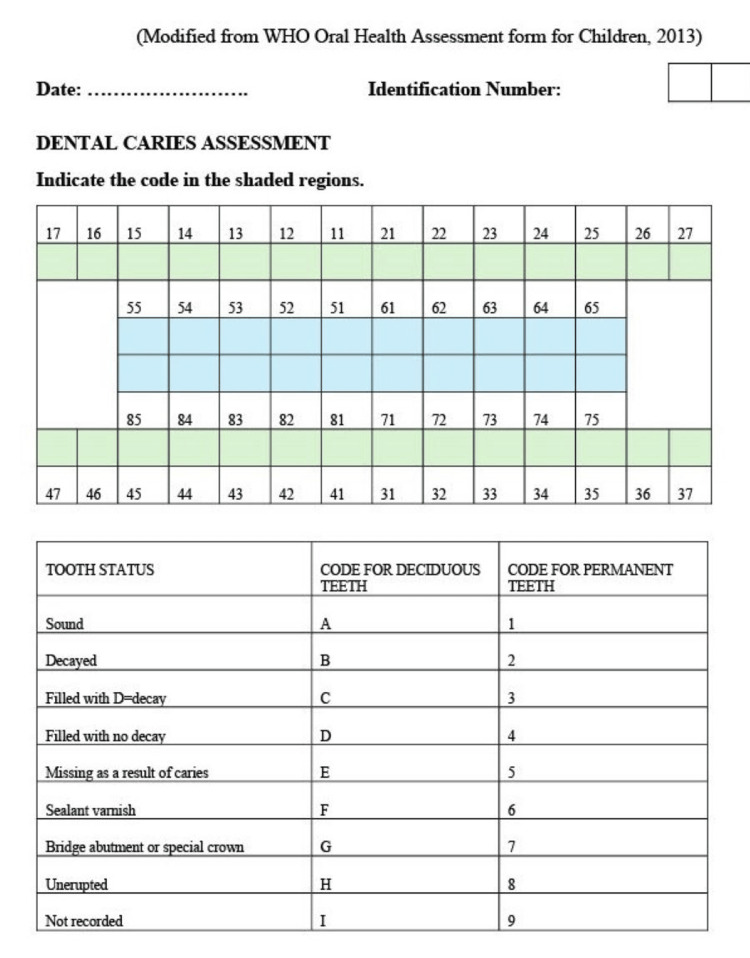
Clinical examination form page 1 Modified from WHO Oral Health Assessment form for Children, 2013 [28]

**Figure 6:**
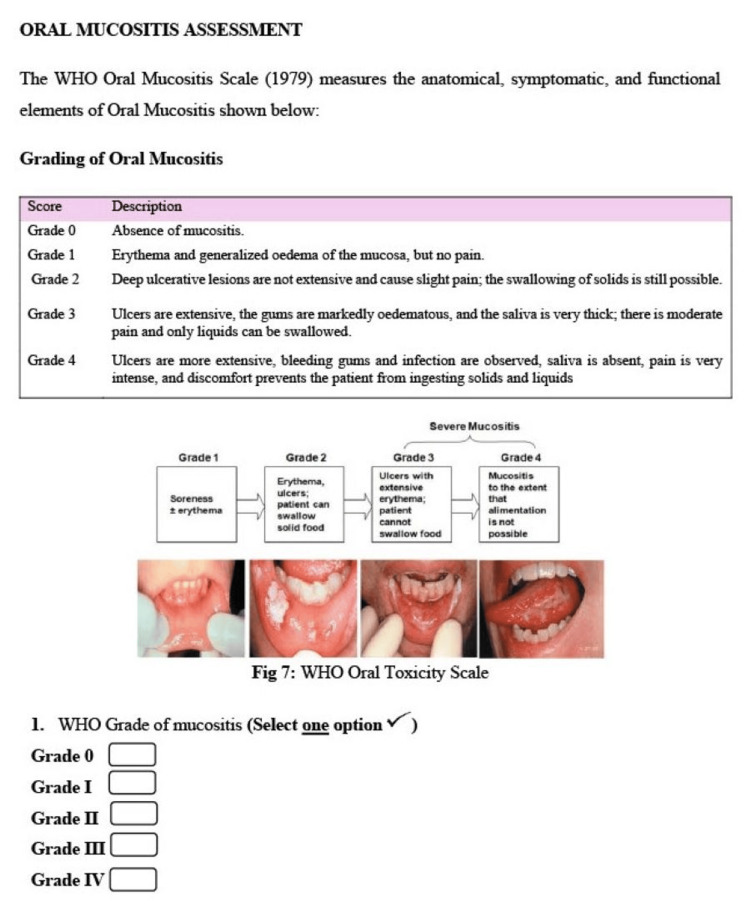
Clinical examination form page 2 Modified from WHO Oral Mucositis Scale (1979) [29]

**Figure 7:**
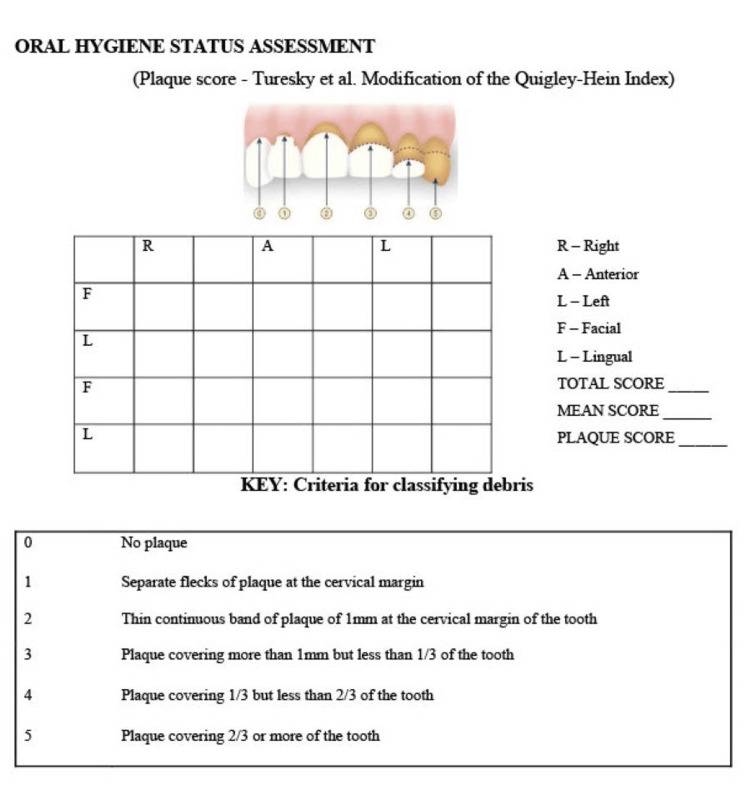
Clinical examination form page 3 Modified from Turesky et al. [31]

## Appendices

Appendix 1: WHO Oral Health Questionnaire 

Appendix 2: Clinical examination form
